# National Association of Dental Examiners

**Published:** 1886-10

**Authors:** 


### National Association of Dental Examiners.

The National Association of Dental Examiners held its fifth session in the Park Theatre, Niagara Falls, commencing Monday, August 2, 1886, President J. Taft in the chair.

The following State Boards were represented: Ohio, by J. Taft, H. A. Smith, and F. H. Rehwinkle; Illinois, by Geo. H. Cushing; Michigan, by A. T. Metcalf, F. W. Clawson, and G. S. Shattuck; California, by S. W. Dennis; Pennsylvania, by S. H. Guilford, W. E. Magill, and E. T. Darby; New Jersey, by Fred. A. Levy; Iowa, by J. T. Abbott; Maryland, by T. S. Waters; Louisiana, by Joseph Bauer; Indiana, by S. B. Brown; Wisconsin, by Edgar Palmer.

Officers were elected for the ensuing year as follows; J. Taft, President; H. A. Smith, Vice-President; F. A. Levy, Orange, N. J., Secretary and Treasurer.

